# Efficient and selective chemical transformations under flow conditions: The combination of supported catalysts and supercritical fluids

**DOI:** 10.3762/bjoc.7.159

**Published:** 2011-09-30

**Authors:** M Isabel Burguete, Eduardo García-Verdugo, Santiago V Luis

**Affiliations:** 1 Department of Inorganic and Organic Chemistry, University Jaume I, Avda. Sos Baynat s/n, 12071-Castellón, Spain

**Keywords:** biphasic systems, continuous flow processes, enantioselective catalysis, immobilized catalysts, polymer-supported systems, supercritical fluids

## Abstract

This paper reviews the current trends in the combined use of supported catalytic systems, either on solid supports or in liquid phases and supercritical fluids (scFs), to develop selective and enantioselective chemical transformations under continuous and semi-continuous flow conditions. The results presented have been selected to highlight how the combined use of those two elements can contribute to: (i) Significant improvements in productivity as a result of the enhanced diffusion of substrates and reagents through the interfaces favored by the scF phase; (ii) the long term stability of the catalytic systems, which also contributes to the improvement of the final productivity, as the use of an appropriate immobilization strategy facilitates catalyst isolation and reuse; (iii) the development of highly efficient selective or, when applicable, enantioselective chemical transformations. Although the examples reported in the literature and considered in this review are currently confined to a limited number of fields, a significant development in this area can be envisaged for the near future due to the clear advantages of these systems over the conventional ones.

## Review

### Introduction

An ideal synthetic process, combining the concepts of classical synthetic chemistry and those of the more recently developed green chemistry, would be one that provides complete conversions of the substrates and that would give place to high (ideally complete) selectivities, with the concomitant generation of only minor (ideally none) amounts of waste and the consumption of the lowest possible amount of energy. This, of course, needs to be associated with the production of relatively large amounts of the desired product with high chemical and economic efficiency and in short time frames [1]. The use of flow systems clearly offers a simple approach towards addressing these last few issues. The main advantages of continuous flow systems using conventional or neoteric solvents have been highlighted in different books and reviews [2–7]. On the other hand, the use of catalytic approaches helps fulfill the other conditions and is always favored from the point of view of green chemistry [8–9]. To facilitate the recovery and reuse of the corresponding catalysts and, simultaneously, to avoid cross-contamination of the products with the catalyst, the use of immobilized systems is clearly preferred [10]. Besides, this is often associated with an increase in the long-term stability of the corresponding catalytic system [11]. For this purpose, immobilization was carried out on different supports. The most usual approach involved the anchoring of the catalytic subunit on a solid support that was either organic (polymeric) [12] or inorganic (zeolites, clays, silicas, etc.) [13]. Nevertheless, other possibilities have been also assayed. These include the use of soluble supports such as dendrimers [14–15], nanoparticles [16], soluble polymers and oligomers [17–18], etc., and the use of liquid phases as the immobilizing media [19–21]. A second method to improve the environmental friendliness of the chemical processes is through the use of neoteric solvents, allowing a reduction, or even elimination, of the use of toxic and environmentally risky organic solvents. In particular, scFs are very well suited for this purpose and, besides, they greatly facilitate the work-up for the isolation of reaction substrates and/or products, for instance by simple expansion of the reaction mixture [22]. Additionally, their tuneable properties provide simple tools for a rapid optimization of reaction parameters and facilitate the development of efficient flow processes [23–25]. Neoteric solvents (scFs, ionic liquids, PEG, etc.) have been considered as “green” alternative solvents due to their properties. However, in order to consider a given process as being more environmentally friendly than the corresponding one run in an organic solvent, the whole process should be evaluated (“cradle-to-grave” evaluation), including an assessment of the energy consumption. Such studies are out of the scope of this paper but they should be kept in mind for their possible implementation.

Supercritical fluids exhibit unique properties that offer the opportunity to manipulate, as required, the parameters of the reaction environment, such as density, viscosity, diffusivity or surface tension, continuously from gaslike to liquid-like properties through the control of pressure and temperature. Thus, for example, varying the temperature and pressure allows manipulation of the density of CO_2_, which determines much of its power as a solvent. At its critical point (31 °C, 74 bar) CO_2_ has a density of 0.46 g·mL^−1^, which, when compared to conventional solvents, may be expected to be associated with relatively weak solvent ability. Increasing pressure increases the density of the CO_2_ for a given temperature. Thus, at pressures of, for example, 120 bar and 40 °C, densities of around 0.7 g·mL^−1^ are typical. The reaction rate and the product selectivity in homogeneous scFs media can be altered by changing these parameters [26–27]. Selected examples of phase equilibrium-controlled chemical reaction kinetics in high pressure CO_2_ have been reviewed elsewhere [28].

Nevertheless, examples exploiting the great potential of the combined use of immobilized catalysts with scFs under continuous flow conditions for chemo- or enantioselective transformations are still limited. The reactions based on the combination of immobilized catalysts and scFs are usually performed under batch conditions [29–30]. The main aim of this paper is to highlight the advantages of the combined use of immobilized catalytic systems and supercritical fluids to develop efficient chemical transformations under flow conditions. This combination not only provides a way to obtain high yields and simple work-up, it can also be used in some cases to implement the selectivity and, when appropriate, the enantioselectivity of the corresponding transformations, although this last aspect is less frequently implemented. The illustrative examples selected for this review have been classified according to four different categories as a function of the reaction type. Our goal is not to present a comprehensive summary of all reactions having been reported for every category of processes but to present some selected and significant cases highlighting the main advantages of this approach.

### 1. Hydrogenation processes

The use of continuous flow hydrogenation protocols employing immobilized metal catalysts has increased significantly over the past few years [31]. Different approaches to the immobilization of catalysts in order to design flow processes in scFs can be mentioned:

#### a) Solid heterogeneous catalysts:

Hydrogenation processes, in particular those based on solid heterogeneous catalysts derived from Ni, Pd, Pt and other noble metals, are of fundamental importance for the industry, and therefore a great effort has been devoted to the detailed study of this kind of processe and to the development of practical protocols for industrial application [32–33].

The use of continuous flow operation conditions in this field has proved to increase the overall efficiency of the studied reactions when compared to the related batch processes [31,34]. One of the main advantages of the scFs is their complete miscibility with gases such as hydrogen. Bringing all reactants into the same phase eliminates the resistance of mass transport across the gas–liquid (or solid) interface. This, in principle, should lead to higher reaction rates. However, the elimination of the gas–liquid mass transport limitation is not the only prerequisite for achieving high global reaction rates. Indeed, for some hydrogenations the reaction occurred faster in two-phase systems (dense solvent and expanded liquid (substrate)) [28]. The reaction rate of the hydrogenation in the presence of scFs is determined, to a great extent, by the phase behavior of the system. On the other hand, supercritical solvents, owing to the reduction of viscosity, can accelerate the adsorption and desorption steps of reactants and products in the catalytic sites. Thereby, a shift from adsorption- or desorption-controlled surface processes to surface-reaction-controlled processes can take place [23,35–37]. Besides, the solvent properties can be easily tuned by a proper selection of pressure and temperature parameters leading to a controlled variation of polarity, dissolution power, etc. All these factors offer good opportunities to perform hydrogenation reactions in scCO_2_ and supercritical hydrocarbons, leading to improved activity and, in some cases, enhanced selectivity.

**Scheme 1:**
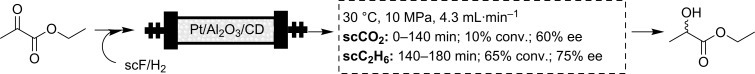
Hydrogenation of ethyl pyruvate.

Some classical examples are provided by the work of Baiker [38–39]. Thus, initial studies focused on the enantioselective hydrogenation of ethyl pyruvate to (*R*)-ethyl lactate over cinchonidine (CD) modified Pt/Al_2_O_3_. The results obtained showed that a dramatic increase in both conversion and enantioselectivity were produced upon changing the solvent from scCO_2_ to scC_2_H_6_ working at 30 °C and 10 MPa, in a flow reactor at 4.3 mmol·min^–1^ (Scheme 1). The poor performance of scCO_2_ was related to the partial catalyst poisoning due to the formation of CO through a water-shift reaction. The use of scC_2_H_6_ was by far superior to the conventional toluene solvent, providing not only an easier separation of products, but also resulting in much higher reaction rates, with an eightfold increase in the resulting turnover frequency (TOF) values.

The conversion (20–60%) and the enantioselectivity (55–75%) were easily tuned by adjusting the H_2_/substrate ratio and the working pressure. The effect of the temperature on the reaction rate was relatively moderate, although, as could be expected, the enantioselectivity significantly decreased with increasing temperatures.

**Scheme 2:**
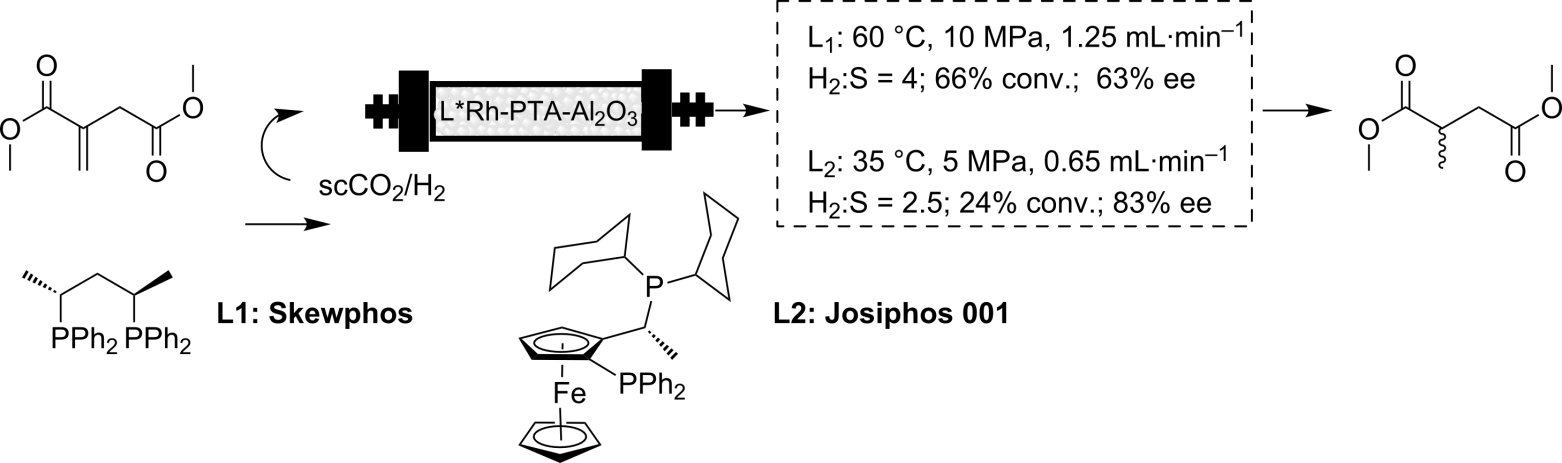
Hydrogenation of dimethyl itaconate.

A second example was provided by the group of Poliakoff [40–41]. In this case, the reaction studied was the hydrogenation of dimethyl itaconate over a chiral Rh catalyst on an inorganic support (Al_2_O_3_) with a phosphotungsten acid linker (Scheme 2). It is important to take into consideration a direct comparison with the work of Baiker, in as much as the catalytic system studied by Baiker is a heterogeneous multiple site system very different from the single site catalyst considered here. In this case, both conversion and enantioselectivity strongly depended on the temperature, the best results being obtained at 60 °C (10 MPa, 1.25 mL·min^−1^, H_2_/substrate ratio = 4). TON values ranged from a minimum of 250 to a maximum of 4600 [moles product]/[moles Rh]. The highest values were obtained at 60 °C and 10 MPa and this corresponds to a TOF of 560 [moles product]·[moles Rh]^−1^·h^−1^ for an eight hour run. Importantly, neither conversion nor enantiomeric excess were observed to degrade over time, under steady-state conditions. Furthermore, with the initial ligand tested (Skewphos) the enantioselectivity achieved was similar to that obtained in conventional solvents (66% ee). Nevertheless, a further screening of a battery of ligands allowed the authors to obtain a system based on the ligand Josiphos, providing a higher enantioselectivity (83% ee at 55 °C) than the corresponding homogeneous system dissolved in scCO_2_ [42].

#### b) Catalysts immobilized in ionic liquids (ILs)

The immobilization of a catalyst in an IL phase has been reported to afford a series of advantages over the use of solid supports: (a) first of all, no modification of the homogeneous catalyst is required for the immobilization, which is a factor that often affects the efficiency of solid-supported systems; (b) the presence of the counter anion can contribute to the activation of the catalyst; (c) the large variety of available ILs can be used for fine-tuning of the overall efficiency of the catalytic system through straightforward modifications in the structure of the IL (anion and cation nature, aliphatic chains, presence of additional functional groups, etc.); (d) finally, the IL phase can significantly contribute to the stabilization of the catalyst [43].

In this case, involving an ILs–scCO_2_ multiphasic system, the CO_2_ phase is intended to favor the delivery of substrates and H_2_ to the IL phase and to facilitate the separation of the final products and the recycling of the IL phase containing the catalyst [44]. The IL–scCO_2_ system shows a specific phase behavior where CO_2_ can dissolve significantly into the IL phase, but no ionic liquid dissolves in the scCO_2_. Thus, the phase behavior of IL–scCO_2_ systems, including the partitioning of organic compounds between both solvents, is essential for developing this type of process, because it determines the contact conditions between scCO_2_ and the solute, as well as the conditions for reducing the viscosity of the IL phase, thus enhancing the mass transfer rate of any catalytic system [45].

**Scheme 3:**
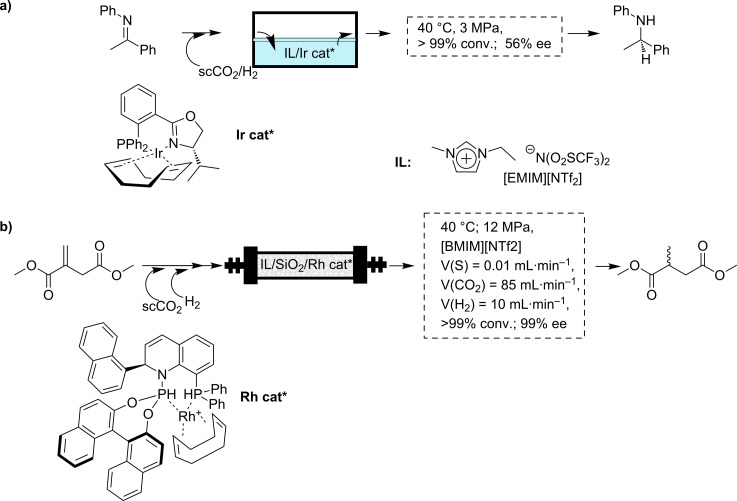
a) Enantioselective hydrogenation of *N*-(1-phenylethylidene)aniline in IL–CO_2_; b) Enantioselective hydrogenation of dimethyl itaconate in SILP–scCO_2_.

The use of this approach is illustrated by the enantioselective hydrogenation of *N*-(1-phenylethylidene)aniline with an Ir catalyst (Scheme 3a) [46–47]. The use of an IL such as [EMIM][NTf_2_] (EMIM = 1-ethyl-3-methylimidazolium) in the absence of scCO_2_ required high H_2_ pressures (10 MPa) to obtain significant conversions (40 °C, 97% conv., 58% ee). In constrast, a quantitative hydrogenation was observed, in the presence of CO_2_, under much milder conditions (40 °C, 3 MPa, >99% conv., 56% ee). As this transformation requires long reaction times (several hours), the initial setup reported by Leitner and coworkers can be described as a repetitive batch system based on successive reaction–extraction cycles (3 h each). This allowed the use of the system for at least seven cycles without any loss in conversion or enantioselectivity. Although this system worked in successive batch cycles, it fully illustrates the potential of biphasic ILs–scCO_2_ systems. Indeed, this system was further implemented by the same authors to obtain a fully continuous process [20].

In this context, a simple approach to implement biphasic ILs–scCO_2_ systems into continuous flow processes is to absorb the ILs on a porous support material (supported IL phase: SILP) [48–51]. This methodology allows simultaneously exploiting the full advantages of a homogeneous biphasic ILs–scCO_2_ system together with those provided by catalysts immobilized onto an insoluble support. In this way, continuous flow catalytic transformations with scFs as a mobile phase to deliver the reactants to the IL phase and to extract the products from there can be easily designed. This approach has been recently used to carry out the asymmetric hydrogenation of dimethyl itaconate. A chiral organometallic catalyst was immobilized on a IL phase absorbed onto a commercial inorganic support, allowing mild reaction conditions and making the use of organic solvents or additional purification steps unnecessary (Scheme 3b). Thus, the commercially available (*S*_a_,*R*_c_)-1-naphthyl-QUINAPHOS in the ionic liquid [EMIM][NTf_2_] gave the best results for the SILP-catalyst system leading to an active catalytic system, stable for 65 h of continuous operation. However, the enantioselectivity could not be kept stable over the entire reaction time. An almost enantiomerically pure product (>99% ee) was obtained for periods of up to 10 h on stream, corresponding to approximately 20,000 catalytic turnovers. A high catalytic efficiency was achieved with this system, reaching a TON value over 100,000 for the chiral transition metal complex, and a productivity of >150 kg product/g of Rh. The system operated with a space time yield (STY) of 0.3 kg·L^−1^ × h. Besides, the catalyst leaching was below the detection limit of 1 ppm [52].

#### c) Catalysts immobilized in other liquid phases

Other unconventional solvents such as fluorous solvents, polyethyleneglycol (PEG) or water were also used in a manner similar to that described above for ILs [19]. An example is the hydrogenation of itaconic acid or acetamido acrylate by means of a chiral Rh catalyst based on a fluorous BINAPHOS ligand. In this case, the substrate and the product are dissolved in water while the liphophilic catalyst is maintained in a scCO_2_ phase, in a so-called inverted biphasic system [53–54]. A regime based on repetitive batch experiments was operated to afford a stable system for five consecutive runs. For this approach, the CO_2_ phase was never depressurized and quantitative yields and excellent enantioselectivities (98.4% ee on average) were achieved (9 cycles, TOF > 200 h^−1^).

### 2. Hydroformylation processes

Hydroformylation is also a reaction of high industrial importance, being one of the main routes to the production of aldehydes [55–56]. In this case, one important factor determining the efficiency of the process, besides the reaction rate, is the control of the selectivity, as either linear or branched aldehydes can be obtained (l/b selectivity). In general, the linear aldehydes are the desired final products for industrial applications. On the other hand, dimerization products can also be obtained along with the desired aldehydes (aldehyde selectivity).

#### a) Solid heterogeneous catalysts

**Scheme 4:**
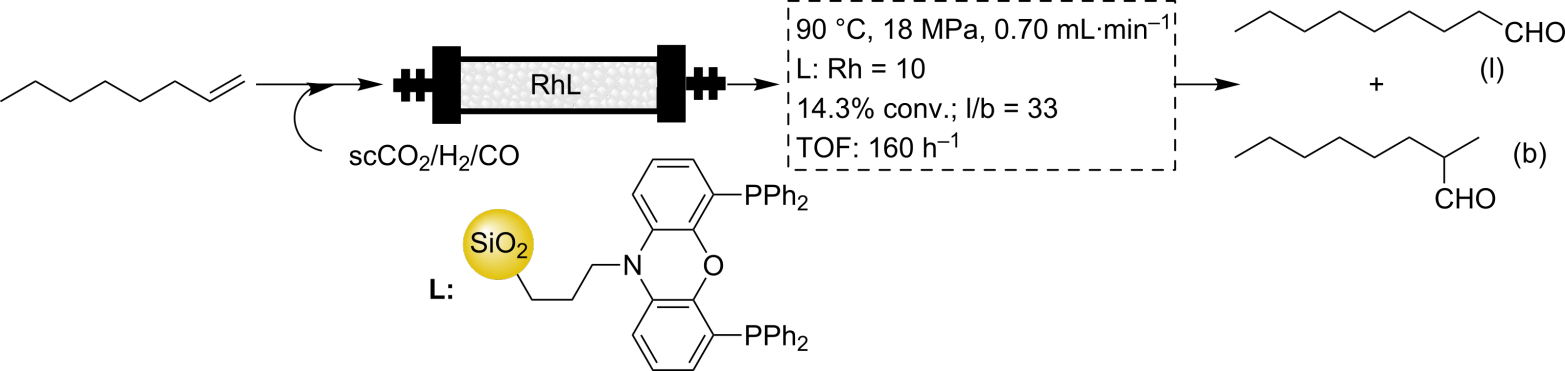
Selective hydroformylation with a silica supported Rh catalyst.

An initial example was provided by Poliakoff and van Leeuwen [57]. A diphosphine ligand immobilized on silica was prepared by a sol–gel approach from *N*-[3-(tri-methoxysilyl)-*n*-propyl]-4,5-bis(diphenylphosphino)phenoxazine (Scheme 4). One remarkable result was that the hydroformylation rate, under flow conditions with scCO_2_ as the carrier phase, was faster than for the batch reaction in toluene, although it was half the rate of the homogeneous system under comparable conditions. This was attributed to the enhanced mass transport properties and the lower viscosity of the medium caused by the presence of scCO_2_. The residual 1-octene was easily separated by controlling the depressurization step and the catalyst performed constantly for 30 h, for six nonconsecutive days.

**Scheme 5:**
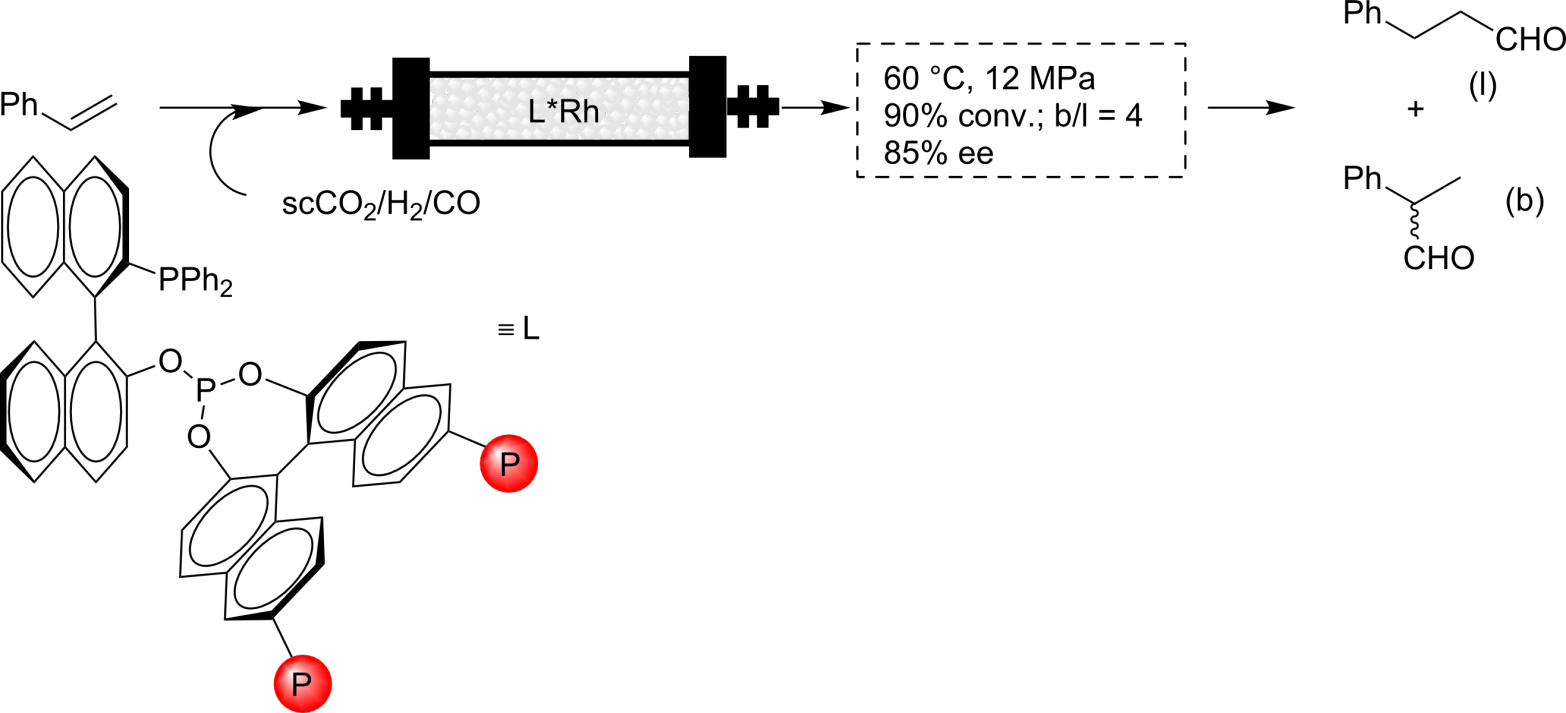
Enantioselective hydroformylation of styrene.

The same authors further explored a variety of different supported ligands for the same reaction, but the results did not improve significantly [58]. The reaction can also be driven towards the branched aldehyde. In this case, the resulting compound has a stereogenic center and an enantioselective process can be developed using a chiral catalyst. An example was reported by Shibahara et al. [59]. For this purpose, a chiral catalyst based on polystyrene-supported (PS) BINAPHOS was employed to form the corresponding Rh catalyst. For volatile alkenes, a direct flow system was used, while for nonvolatile substrates scCO_2_ was used as the carrier phase. Thus, for styrene a ca. 50% conversion was achieved with an 80:20 b/l ratio and 85% ee (Scheme 5).

#### b) Catalysts immobilized in ionic liquids (ILs)

The immobilization of a Rh hydroformylation catalyst in [BMIM][PF_6_] has been reported by Cole-Hamilton to produce a significant decrease in the aldehyde selectivity, relative to toluene, while retaining the conversion and the l/b selectivity [60–61]. Changing to a scCO_2_–IL biphasic system produced a decrease in the conversion but the aldehyde selectivity was recovered and, more importantly, the l/b selectivity experienced a threefold increase. It has been considered that the main role of scCO_2_ is to reduce the residence time of the product aldehyde in the catalyst solution and to protect it from further reaction. This can be associated with the rapid diffusion of the product from the IL phase to the scCO_2_ phase. A comparison with Co-based commercial catalysts clearly favors the new system in terms of productivity, although other factors such as robustness, long-term productivity and operational costs should be also considered and have not yet been analyzed. The system has been further improved through the use of different Xantphos-related ligands containing imidazolium subunits to favor the solubility in the IL phase [62]. A system involving the immobilization of the catalyst in a film of an IL supported on silica (SILP) has also been described with either scCO_2_ [63] or in the gas phase [64]. The results obtained for the hydroformylation in SILP–scCO_2_ revealed the achievement of higher rates, as compared with the bulk-IL–scCO_2_ system, reaching TOF values of 800 h^−1^ and more than threefold improved STYs. Statistical methods and a thorough study of the phase behavior were used to optimize the system. Optimum conditions were located just below the critical point of the mixture in an expanded liquid phase regime. High reaction rates corresponding to TOF values of 500–800 h^−1^ could be maintained for at least 40 h of continuous flow operation (TON > 20000) with low catalytic leaching [65].

### 3. Miscellaneous chemocatalystic processes

Reactions catalyzed by heterogeneous acids in supercritical fluids have been widely studied under flow conditions [66–68]. This approach is attractive because it combines acid heterogeneous catalysts, as an environmentally and economically acceptable alternative to conventional homogeneous catalysts such as H_2_SO_4_ and HF, with the advantages of scFs. Thus, simple fixed-bed reactors can be developed for carrying out continuous flow processes. Furthermore, the catalyst lifetime can, in many cases, be dramatically improved under supercritical conditions, owing to reduced coking [69–70].

The selective monoprotection of 1,*n*-terminal diols is a characteristic example of how mono-substituted ethers can be selectively prepared versus the bis-substituted ones [71]. The use of an acid catalyst (Amberlyst 15) allows the formation of the corresponding mono-ethers by reaction of 1,6-hexanediol and other diols with simple alcohols under flow conditions (scCO_2_, 150 °C, 20 MPa, 1.15 mL·min^−1^). One of the most interesting results found was the observation that, at 150 °C, the selectivity of the reaction can be switched by a proper adjustment of the pressure. Thus, at 5 MPa the bis-ether predominates over the mono-ether by a factor of 20, while the reverse selectivity is achieved at 20 MPa (mono-/bis-ether = 9). This was shown to be related to the phase state of the reaction mixture. Thus, those results confirmed that it is possible to control the alkylation selectivity by a simple tuning of the properties of the supercritical fluid.

**Scheme 6:**
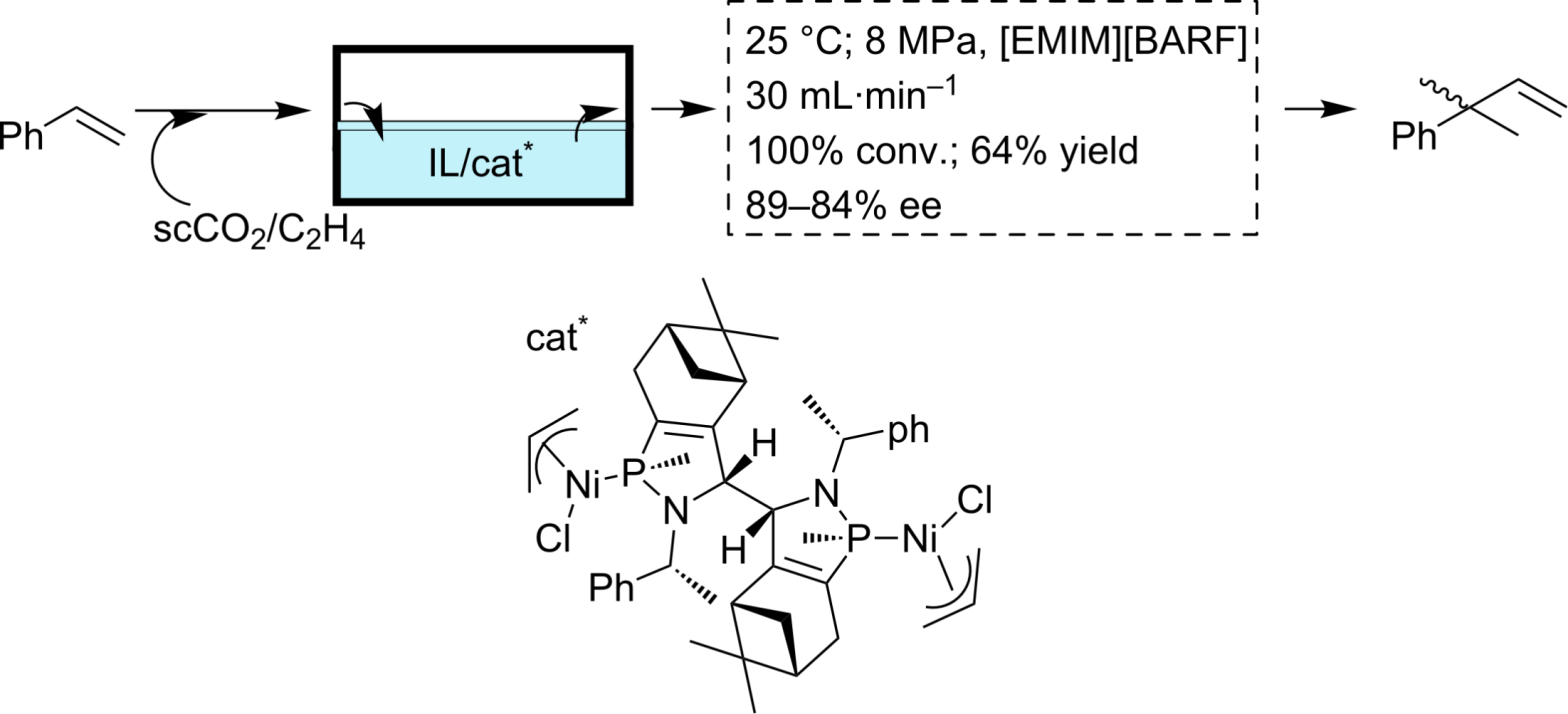
Enantioselective hydrovinylation of styrene.

A second example involves the hydrovinylation of styrene using the Wilke’s catalyst immobilized on an IL (Scheme 6) [72]. The work under continuous flow conditions with scCO_2_ afforded good conversions and selectivities (25 °C, 8 MPa, 100% conversion, 64% yield, 89–84% ee). The presence of the IL phase facilitated the activation of the organometallic catalyst, while the combination with scCO_2_ allowed a significant decrease in the viscosity of the IL solution, improving the mass transfer. Finally, the work under continuous flow conditions avoided the deactivation of this sensitive catalyst, as observed during its recycling in the batch mode. The increased long-term stability of the catalyst was associated with the low stability of the active species in the absence of the substrate and this could be avoided or minimized by continuous operation in a flow reactor. Thus, more than 60 h of operation time could be achieved moving from batch to continuous flow.

**Scheme 7:**
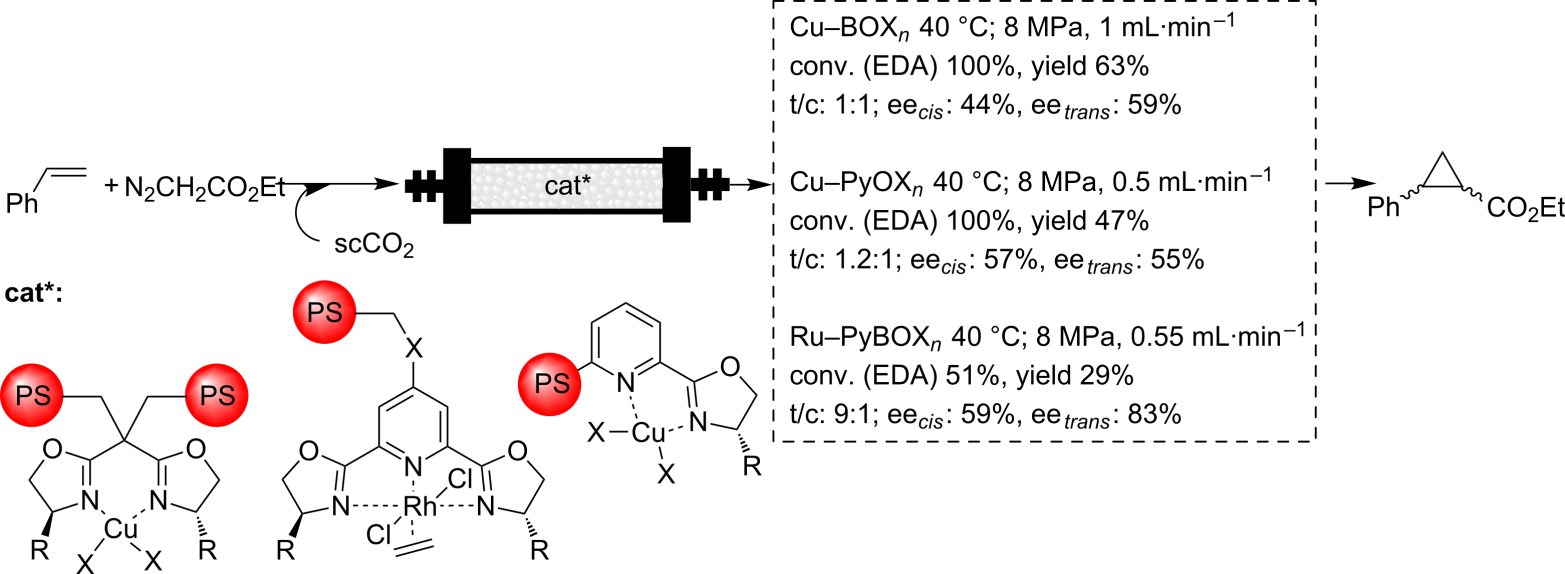
Enantioselective cyclopropanation of styrene catalyzed by supported Cu–BOX, Cu–PyOX and Rh–PyBOX catalysts.

Finally, the remaining examples concern our own work on the enantioselective cyclopropanation with supported copper–bisoxazoline (Cu–BOX) or copper-pyridineoxazoline (Cu–PyOX) complexes and related systems. The oxazoline ligands can be introduced in polystyrene–divinylbenzene (PS–DVB) matrices either by grafting, by reaction with chloromethyl groups of preformed resins, or by polymerization of the corresponding bisoxazolines or pyridineoxazolines containing polymerizable vinylic fragments [73–74]. The transformation of those BOX or PyOX moieties into the corresponding Cu complexes allows their use as catalysts for the enantioselective cyclopropanation of styrene (Scheme 7). Similar approaches allow the preparation of polymers containing PyBOX subunits, from which the corresponding Rh complexes can be formed and studied for the same reaction [75]. The preparation of this kind of catalyst in the form of monolithic polymers inside stainless steel columns is very well suited to work under flow conditions [76–77]. Both catalysts were studied in flow conditions with scCO_2_ (40 °C, 8 MPa), with conventional solvents and under solvent-free conditions [74,78–79]. One of the main outcomes of the results obtained was the observation of a significant improvement in the productivity when going from conventional to supercritical conditions within the same reactor. Thus, for instance, when considering the total production per volume for a given time, the value obtained, working in continuous flow and using the Cu–PyOX system as the catalyst, was higher for scCO_2_ than that achieved for methylene chloride (DCM) (1402 g·L^−1^ versus 836 g·L^−1^). It is noteworthy that this higher volumetric productivity was obtained in a shorter time, 165 min for scCO_2_ compared with 540 min for DCM. Hence, in scCO_2_ up to 1.5 mmol of cyclopropanes per hour can be obtained, whereas lower values, around 0.13–0.21 mmol·h^−1^, were achieved in DCM. More interestingly, in all cases the conversion, chemoselectivity, selectivity and enantioselectivity were comparable or slightly superior to those found for the work under batch conditions with the same catalysts. The results obtained also revealed that one additional advantage of the work under flow conditions with scFs is the easy and fast optimization of the system through a proper control of reaction parameters, such as pressure, temperature and flow.

### 4. Biocatalytic processes

The proper use of catalytic [8] and engineering [80] methodologies can improve significantly the energetic and synthetic efficiencies, as well as reaction selectivity, while reducing the production of concomitants. In this regard, biocatalysis-based transformations have an enormous potential, since they are able to increase stereo-, chemo- and regioselectivities for a vast number of chemical transformations [81–82]. Besides, a great variety (more than 13,000) of enzyme-catalyzed reactions have been successfully demonstrated at the laboratory scale, offering clear advantages for the synthesis of enantiopure fine chemicals over any other kind of catalysts [83–84]. As a result, the chemical industry is currently exploring the great potential of biocatalysis to manufacture both bulk and fine chemicals [85–86]. Although many of the reported examples involve the use of batch conditions, different examples of continuous biocatalytic processes have been described with conventional solvents [87]. The use of biocatalytic flow processes under unconventional conditions presents additional advantages [88]. Indeed, flow systems operating above atmospheric pressure enable the possibility of using fluids above their boiling point just by including a retention valve at the exit of the reactor. This facilitates the practical use of this kind of reactor in superheated solvents [89] or with supercritical fluids, either in scCO_2_ [90–91] or also in non-scCO_2_ supercritical fluids [92].

#### a) Solid supported biocatalysts

**Scheme 8:**
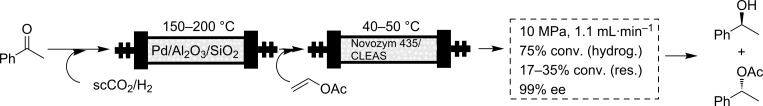
Continuous hydrogenation of acetophenone coupled with the kinetic resolution of the product.

Nowadays many enzymes are commercially available immobilized on different solid supports and have found to be compatible with supercritical conditions [88]. In general, most of the examples are related to the preparation of nonchiral products, but the use of biocatalysts is even more interesting when the synthesis of chiral compounds is involved. Different examples can be found, in this regard, in the literature. The most common transformations are the kinetic resolution (KR) of secondary alcohols. For example, Matsuda has reported the use of the commercially available supported lipase Novozym 435 for the kinetic resolution of aromatic and nonaromatic substrates using scCO_2_ (42 °C, 13 MPa) [93]. The space time yield was improved by a factor of 400 under flow conditions as compared to that of the corresponding batch processes. The enantioselectivity of the process was excellent and enantiomeric ratio values (*E*) larger than 1800 were obtained. The system maintained its activity and selectivity over at least three days of continuous use, allowing the resolution of 221 g of (*±*)-1-phenylethanol to the (*S*)-1-phenylethanol (99% ee) and the corresponding (*R*)-acetate (99% ee) with just 1.73 g of supported enzyme. An interesting flow system is that reported by Poliakoff and Sheldon involving two sequential reactions (Scheme 8) [94]. In that case, the first step was the continuous hydrogenation of acetophenone to afford the racemic phenylethanol with Pd supported on SiO_2_/Al_2_O_3_ as the catalyst and scCO_2_ as the reaction medium (150–200 °C, 10 MPa, 1.1 mL·min^–1^, H_2_:substrate ratio = 4). The racemic alcohol (75% conversion for the first step) was directly fed to a second catalytic reactor containing the supported biocatalyst to carry out the kinetic resolution (40–50 °C, Novozym 435 or CLEAS as the immobilized enzyme). Although only moderate conversions were achieved for the second step (17–35%) the corresponding acetate was obtained in high enantiomeric purity (ca. 99%).

#### b) Biocatalysts immobilized in ionic liquids (ILs)

**Scheme 9:**
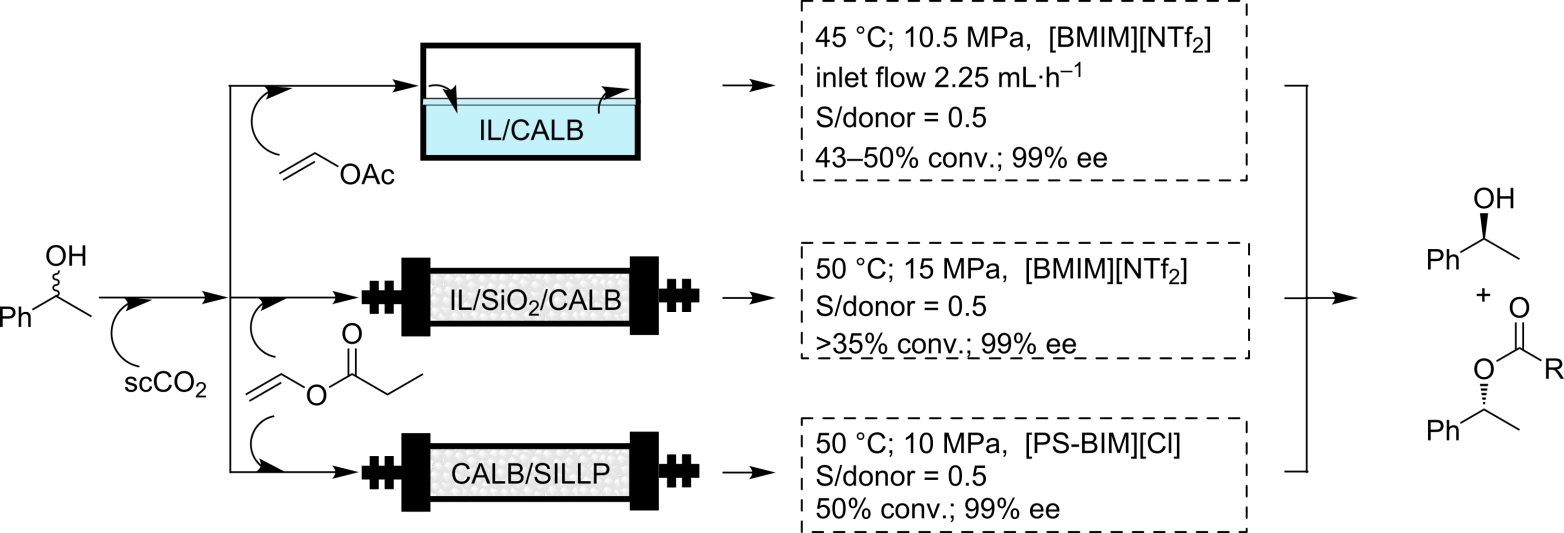
Kinetic resolution of phenylethanol using CALB immobilized in ILs and supported ILs.

The discovery of the possibility to immobilize biocatalysts in ionic-liquid phases has been a recent breakthrough that can be compared with the progress made by the use of enzymes in organic solvents [95–96]. One of the main advantages reported for the enzyme–IL system is the enhanced stability found for the biocatalysts especially in water-immiscible ILs [88,97]. The observed stabilization of enzymes in water-immiscible ILs was attributed to their inclusion in hydrophilic gaps of the network, where they are surrounded by a strong ionic net. The extremely ordered supramolecular structure of the ILs in the liquid phase may be able to act as a “mould”, maintaining the active 3D structure of the enzyme and avoiding unfolding [88]. The combination of supporting the biocatalyst in an IL (either dissolved or suspended) with the use of a scF for delivering the substrates to the IL phase and to extract the final products from there, allows one to take full advantage of the above mentioned benefits and to develop continuous flow biocatalytic systems in which the IL phase acts as the stationary phase and the scF becomes the mobile phase [98]. Pioneering work in this field was carried out by the groups of Lozano and Leitner and Reetz [99–100]. Both groups studied the kinetic resolution of phenylethanol with *Candida Antarctica lipase B* (CALB) as the biocatalyst (Scheme 9). In the case of the work by Leitner and Reetz, the CALB enzyme was suspended in a bulk IL phase. At 45 °C and 10.5 MPa and using a substrate:donor ratio of 0.5 they achieved conversions of 43–50% with 99% ee values for the acetate. In a further improvement of the system, the same authors reported the coupling of the resolution step with the continuous separation of the two products formed based on their different solubilities in the scCO_2_ phase [101]. Reetz et al. also extended this methodology to a biphasic polyethyleneglycol (PEG)/scCO_2_ system [102]. An excellent conversion was maintained for at least 25 h. After a short induction period, the amount of product was almost identical to the amount of substrate and the system operated with a constant high activity of 5700 mmol·min^−1^·g^−1^ corresponding to a space time yield of 0.1 kg·L^−1^·h^−1^. Since an excess of vinyl acetate was used, all of the PEG 1500 was also acylated.

A different approach was developed by Lozano et al. In their case, a thin layer of an IL phase containing the enzyme was created on the surface of silica and the resulting material was used in a packed-bed reactor, using vinyl propionate as the acyl donor (50 °C, 15 MPa, substrate:donor ratio 0.5, conversion >35%, 99% ee for the product). It is worth mentioning that, in the last case, the enzyme maintained an excellent activity and a reasonable stability even at 150 °C [103], which reveals the important stabilization associated with the combination of the biocatalyst with the IL phase [99]. These authors studied in detail the influence of the support and the IL used, as well as the effect of the previous adsorption of the enzyme onto a modified silica surface [104]. A partly related approach is the immobilization of the lipase onto a covalently supported ionic liquid-like phase (SILLP) [105–106]. Those SILLPs are prepared by functionalization of PS–DVB surfaces with IL-like (imidazolium) moieties [107–108]. Experimental results have shown that the resulting surfaces have similar physico-chemical properties to those of the corresponding bulk ILs [109]. CALB supported on those SILLPs was shown to be an efficient and very stable catalyst for the continuous flow synthesis of citronellyl propionate in scCO_2_. The presence of a high IL/enzyme ratio is reflected in a high stabilization of the CALB in those systems. Thus, the process was carried out at 80 °C with yields of 93% for more than 10 h without any appreciable deactivation of the enzyme. Considering enantioselective transformations, the dynamic kinetic resolution (DKR) of phenylethanol was studied under continuous flow in scCO_2_. This was easily achieved by the combination of a supported enzyme (Novozym 435) and a supported acid catalyst, both covered by a thin layer of an IL. This has allowed the development of the continuous chemoenzymatic dynamic kinetic resolution of the corresponding racemic alcohols with yields of up to 80% and enantioselectivities of about 91–98% [110–111]. Essentially enantiopure products with at least 80% yields were obtained with supported CALB as the biocatalyst.

Similarly, the synthesis of DKR phenylethanol can be performed by the combination of the two catalysts in a “one pot” single columnar minireactor. This was packed with a mixture of zeolite CP811E-150 as the acid catalyst for the racemization and CALB supported on a SILLP based on bead-type PS-DVB resins [106]. Good results were obtained when the zeolite catalyst was coated with a small amount of an IL. This follows the same trend observed in the case of SILPs. Nevertheless, contrary to the observations obtained with the use of commercial immobilized CALB (Novozym 435), no additional IL coating of the biocatalyst was required for the stabilization of the enzyme in the case of CALB–SILLPs. This clearly highlights how SILLPs are able to efficiently stabilize CALB against deactivation by scCO_2_ or the presence of acidic catalysts. The best results were obtained by an appropriate control of the flow rate, and the DKR of *rac*-phenylethanol could be carried out with excellent yields and enantioselectivities (92%, >99.9% ee for the *R*-ester). The systems based on SILLPs have shown a remarkable stability, with consistent results for at least 17 days of continuous operation.

## Conclusion

The development of flow processes using scFs in combination with catalysts supported either on solid or liquid phases (ILs, PEG, fluorous phases, etc.) presents a large potential for enantioselective reactions. In the examples shown, the activity, selectivity and enantioselectivity of the processes was modulated through the proper adjustment of the reaction parameters, such as pressure, temperature, flow, and the nature of the scF, etc., allowing for a rapid optimization of the process. An adequate design of the support (the nature of the solid matrix or the chemical nature of the IL for instance) was used advantageously to improve the overall efficiency of the system. A significant improvement in productivity was obtained, in general, under flow conditions. One of the reasons for such improvement is the increase in mass transfer provided by the scF and associated with the enhanced diffusion of substrates and reagents through the interfaces favored by the scFs. The long-term stability of the catalysts achieved under those conditions was also an additional factor to increase the final productivity. This last factor has often been related to the removal of the work-up steps associated with the recovery of the catalyst, which are frequently sensitive to humidity, air, or changes in temperature/pressure under batch conditions.

Up to now, the examples reported in the literature for enantioselective processes under flow conditions with scFs are confined to a limited number of fields, but considering the clear advantages of the use of these methodologies over the conventional ones, one should expect a significant development in this area for the near future, including the development of industrial applications.
